# Field phenotyping of grapevine growth using dense stereo reconstruction

**DOI:** 10.1186/s12859-015-0560-x

**Published:** 2015-05-06

**Authors:** Maria Klodt, Katja Herzog, Reinhard Töpfer, Daniel Cremers

**Affiliations:** 1 Department of Informatics, Technische Universität München, Boltzmannstraße 3, 85748 Garching, Germany; 2 Julius Kühn-Institute - Federal Research Centre for Cultivated Plants, Institute for Grapevine Breeding Geilweilerhof, 76833 Siebeldingen, Germany

**Keywords:** Image analysis, Depth maps, Image segmentation, Digital leaf area, Fruit-to-leaf ratio, Leaf classification, *Vitis vinifera*

## Abstract

**Background:**

The demand for high-throughput and objective phenotyping in plant research has been increasing during the last years due to large experimental sites. Sensor-based, non-invasive and automated processes are needed to overcome the *phenotypic bottleneck*, which limits data volumes on account of manual evaluations. A major challenge for sensor-based phenotyping in vineyards is the distinction between the grapevine in the foreground and the field in the background – this is especially the case for red-green-blue (RGB) images, where similar color distributions occur both in the foreground plant and in the field and background plants. However, RGB cameras are a suitable tool in the field because they provide high-resolution data at fast acquisition rates with robustness to outdoor illumination.

**Results:**

This study presents a method to segment the phenotypic classes ‘leaf’, ‘stem’, ‘grape’ and ‘background’ in RGB images that were taken with a standard consumer camera in vineyards. Background subtraction is achieved by taking two images of each plant for depth reconstruction. The color information is furthermore used to distinguish the leaves from stem and grapes in the foreground. The presented approach allows for objective computation of phenotypic traits like 3D leaf surface areas and fruit-to-leaf ratios. The method has been successfully applied to objective assessment of growth habits of new breeding lines. To this end, leaf areas of two breeding lines were monitored and compared with traditional cultivars. A statistical analysis of the method shows a significant (p <0.001) determination coefficient R ^2^= 0.93 and root-mean-square error of 3.0%.

**Conclusions:**

The presented approach allows for non-invasive, fast and objective assessment of plant growth. The main contributions of this study are 1) the robust segmentation of RGB images taken from a standard consumer camera directly in the field, 2) in particular, the robust background subtraction via reconstruction of dense depth maps, and 3) phenotypic applications to monitoring of plant growth and computation of fruit-to-leaf ratios in 3D. This advance provides a promising tool for high-throughput, automated image acquisition, e.g., for field robots.

**Electronic supplementary material:**

The online version of this article (doi:10.1186/s12859-015-0560-x) contains supplementary material, which is available to authorized users.

## Background

Grapevines (*Vitis vinifera* L ssp. *vinifera*) are highly susceptible to several fungal diseases (e.g. powdery mildew and downy mildew) and require substantial effort to protect the plants. This susceptibility is the major reason for extended grapevine breeding activities around the world which aim at selecting new cultivars with high disease resistance and high quality characteristics [1]. Representing a perennial woody crop plant, grapevine phenology, analysis of growth habits and yield traits can only be evaluated in the field.

The analysis of growth habits is an important aspect in viticulture for site specific canopy management. The aim is to improve grape yield and wine quality [2]. Three factors in particular describe the relationship between canopy structure, light microclimate and grape quality: 1) the geometrical dimensions of the canopy, 2) the foliage density as an indicator of leaf exposure to sunlight, and 3) the bunch exposure to sunlight [3]. The determination of grapevine architecture, e.g., canopy surface area including vigor during the vegetative period and the respective position of organs (leaves, stems and bunches), can be used for dynamic characterization of breeding material and site-specific canopy management. The overall aim is to achieve an optimal canopy microclimate, especially for the grape cluster zone, i.e., minimal shade and aerated conditions [2]. In addition, a balanced ratio between vegetative (shoots and leaves) and fruit growth is important to avoid excess or deficient leaf areas in relation to the weight of the fruit [2]. This fruit-to-leaf characterization requires quantifications of the canopy surface dimension and the grapes.

In traditional breeding programs, phenotyping of grapevines is performed by visual inspection. Thus, data acquisition is time consuming, laborious, and the resulting phenotypic data are the subjective assessment of the personnel in charge. Traits can be described with OIV descriptors [4] or the BBCH scale [5]. The OIV descriptor 351 [4] is used to classify grapevine vigor to five categories (1 = very weak; 3 = weak; 5 = medium; 7 = strong; 9 = very strong vigor). Accurate characterization of grapevine growth from a large number of cultivars (viticulture) or breeding material (grapevine breeding) requires simple, fast and sensor-based methods which are applicable from a moving platform for high-throughput data acquisition [6]. Numerous indirect and non-invasive methods have been studied to characterize grapevine foliage directly in vineyards [3,7-9]. Most of the studies are based on costly sensor techniques, e.g. electromagnetic scanners [3], ultrasonic sensors [10], laser scanners [9], infrared sensors [11], fish-eye optical sensors [11-13] or model based strategies [7]. Some of these methods correlate with destructive sampling from direct measurements taken with a leaf area meter [12,14,15]. Electromagnetic and laser scanners directly obtain 3D point clouds of a scene, however provide no volumetric and surface information. Other active sensors include time-of-flight and structured light sensors, which are specialized for indoor environments. These types of sensors can have difficulties in scenes with bright illumination or large distances as often occur in outdoor environments [16].

Image analysis provides a promising technique for non-invasive plant phenotyping [17]. RGB cameras are a practical sensor for usage in the field because they are portable, provide fast data aquisition and are suitable for outdoor illuminations. However, only a few studies exist on automated approaches for monitoring grapevine growth habits directly in vineyards using low-cost consumer cameras [6,8,18]. Color is an important indicator for vegetation and can be used to detect leaves in images [19]. When the foreground plant should be segmented from the background however, a color image alone is often not sufficient. This is due to the fact that the foreground plant and the background containing the field and other plants usually have the same color distributions. The use of single RGB images then requires elaborate installation of artificial backgrounds in the field, to determine the canopy dimensions from grapevines [8]. Furthermore, the image projection process can create size distortions in the 2D image plane, e.g. if some parts of the plant are closer to the camera than others [6,18].

From this point of view, additional 3D information can help to increase the precision of phenotypic data and to eliminate the background automatically [6]. Full 3D models of plants from images were computed in [20] and [21] for segmentation of leaves and stems. In [22] an image based method for 4D reconstruction of plants based on optical flow is introduced. Depth maps, providing 3D information in the image domain, have been used for the determination of leaf inclination angles [23] and bud detection [6]. Stereo reconstruction methods have been intensively studied in the field of computer vision [24,25]. Respective methods can be divided into sparse reconstruction where 3D point clouds are computed [26,27], and dense reconstruction which aims at computing surfaces [24]. The use of sparse 3D information yields little information in homogenous image regions and can result in inaccurate classification results for phenotyping as has been shown in [6]. Thus, dense 3D surfaces are essential for a reliable detection of the foreground (i.e., the grapevine) and elimination of redundant background (i.e., the field).

**Figure 1:**
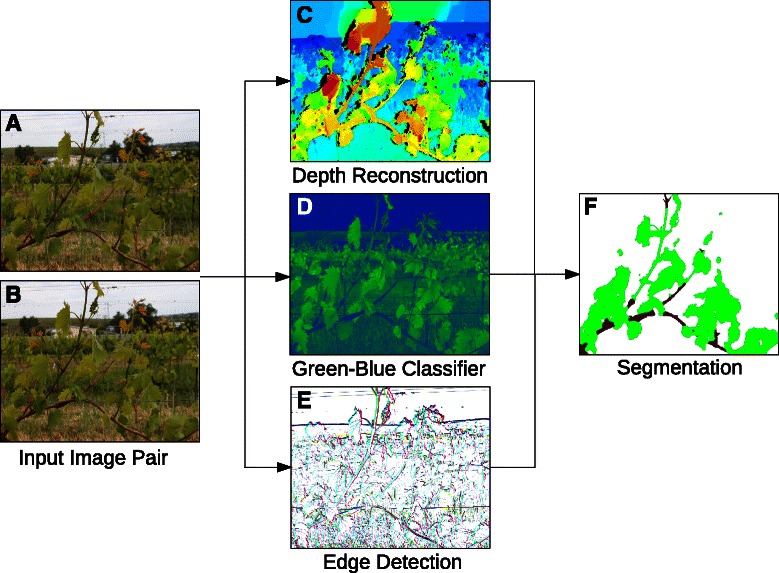
Workflow of the proposed image-based phenotyping method. Stereo image pairs **(A, B)** are captured from a moving platform in a vineyard. From these images the method computes a dense depth map **(C)**, a color classification based on the the green and blue color channels **(D)**, and an edge detector **(E)**. These features are used to segment the image domain to ‘leaf’, ‘stem’ and ‘background’ **(F)**. The image segmentation allows for objective computation of phenotypic indicators like the visible leaf area and fruit-to-leaf ratio.
- The method is robust for background subtraction in particular, because of the use of dense depth maps from stereo reconstruction. We developed a stereo reconstruction algorithm that is particularly suitable for the fine-scaled features of plant geometry. This avoids elaborate application of artificial backgrounds during image acquisition, even for images where the foreground and background have similar color distributions.
- Using a standard consumer camera, data acquisition is fast, simple and portable. The method is thus particularly practical for phenotyping of grapevine, which can only be evaluated directly in the fields.
- The method has been successfully applied to non-invasive and objective monitoring of grapevine growth and computation of fruit-to-leaf ratios in 3D. Furthermore, we show how growth habits of new breeding lines are classified by comparison to known cultivars.

This study presents a novel approach for non-invasive, fast and objective field phenotyping of grapevine canopy dimensions. The method is based on dense depth map reconstruction, color classification and image segmentation from image pairs. An overview of the method is shown in Figure 1. The main contributions of this study are the following:

- We present a method to robustly segment RGB images of grapevine to the phenotypic classes ‘leaf’, ‘stem’, ‘grapes’ and ‘background’. The segmentation is based on color and depth information. The results allow for objective phenotypic assessment of large data sets which yields a step towards overcoming the phenotypic bottleneck.

## Results and discussion

This study presents a method for objective computation of phenotypic traits of grapevine from RGB images captured in vineyards. The method is based on 1) the automated elimination of the background from RGB field images by using dense stereo reconstruction, 2) the automated detection of leaves, grapes and stem in the foreground, and 3) the quantification of the visible leaf area. The following shows phenotypic applications to monitoring of grapevine growth and computation of fruit-to-leaf ratios in 3D. It furthermore shows how growth monitoring enables the classification of new breeding lines by comparing their growth habits to known cultivars.

### Monitoring of grapevine growth

In the following, we show how the presented method can be applied for the analysis of growth habits of breeding material with unknown properties. Objective assessment is achieved by monitoring leaf areas over time and comparing them to traditional cultivars that are used as a reference. To this end, we monitored two breeding lines and sample plants of two traditional cultivars with different growth habits (‘Riesling’ with medium shoot growth and ‘Villard Blanc’ with weak shoot growth) during a season.

**Figure 2:**
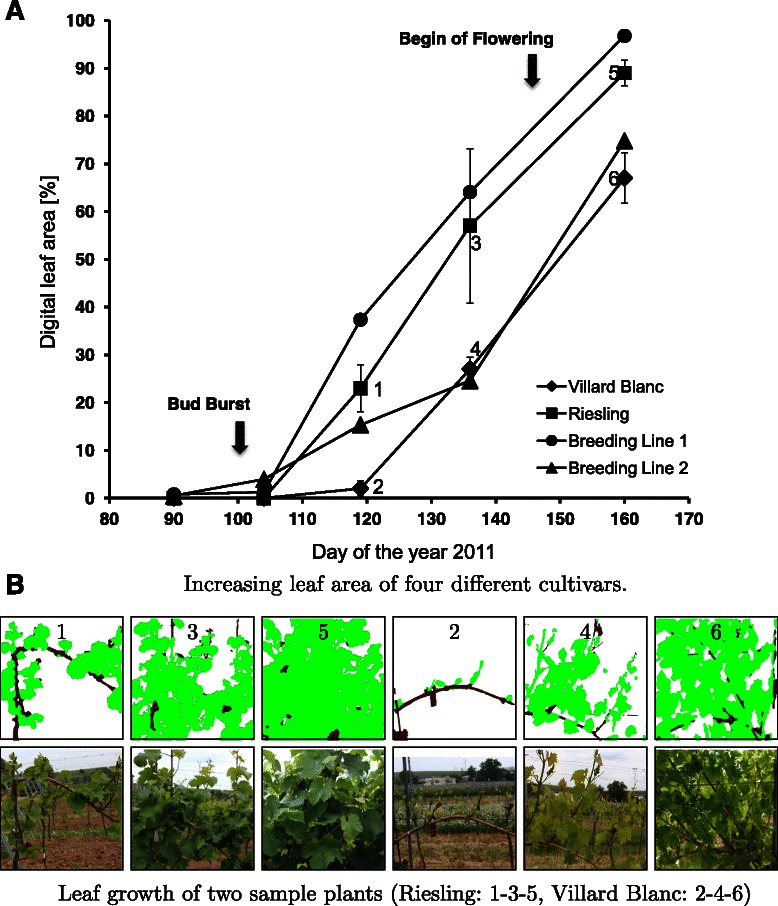
Image-based monitoring of grapevine growth. Two breeding lines with unknown growth characteristics are compared to the known cultivars ‘Riesling’ and ‘Villard Blanc’. The progression graph **(A)** shows increasing leaf areas for the four cultivars during the vegetative growth phase. Numbers were labeled with reference to two of the sample plants that were monitored **(B)**.

Figure 2A shows the computed leaf area per breeding line and the average and standard deviation of leaf areas for the two traditional cultivars.Standard deviations were only computable for the reference cultivars ‘Riesling’ and ‘Villard Blanc’. From both cultivars three plants were used respectively as biological repetitions at each time point. From the investigated breeding lines only one single plant was available per time point. This is due to the fact that only one biological repetition is available from the breeding lines and thus, no standard deviation was calculated. Figure 2B shows the images of two sample plants that were monitored at multiple time points. As expected, none of the investigated genotypes displayed a detectable vegetative growth before bud burst. For all genotypes, an increasing leaf area can be observed between the 90th day and the end of the experiment on day 160. Differences in the percentage were used for objective scoring of plant growth.

We observed a ten times faster growth of ‘Riesling’ compared to the cultivar ‘Villard Blanc’. As also shown in Figure 2A, the genotypes offer the major differences in plant growth at day 120. Two weeks later two groups were observed: group 1 consisting of ‘Riesling’ and breeding line 1; and group 2 consisting of ‘Villard Blanc’ and breeding line 2. At day 160, breeding line 1 almost displayed the maximum feasible leaf area of 100 %. This genotype also exhibited the fastest growth during the entire experiment. The second breeding line grew at a slower rate and had a smaller digital leaf area at day 160 and thus seems to be more related to ‘Villard Blanc’.

These results show that the presented method enables an objective distinction of cultivars. This is essential for a reliable identification of subtle differences in visible canopy dimensions and for the objective, comparable characterization of breeding material with unknown phenotypic properties, e.g. on different field sites or different vineyard management conditions. The images were captured at different time points until the first canopy reduction. This enables an objective monitoring of the vegetative growth in a defined time scale. We observed two groups of growth habits which facilitate an objective evaluation of the investigated breeding lines.

Thus, the method is also a promising tool for the identification of genotype specific differences in growth rates or for efficiency analysis of plant protection efforts. This kind of fast, objective and comparative monitoring of plant development further enables the study of growing dynamics with respect to climatic influences or soil properties.

### Computation of fruit-to-leaf-ratios in 3D

The segmentation of the images to ‘grapes’, ‘leaves’ and ‘background’ allows for an investigation of bunch positions in the canopy. This involves the analysis of which grape bunches are overlaid with leaves, the amount of bunch exposure to sunlight and whether a grapevine site shows a well-balanced fruit-to-leaf ratio.

**Figure 3:**
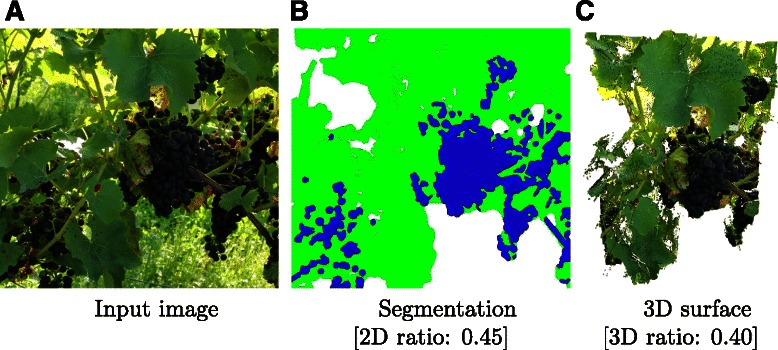
Generalization to the phenotypic class ‘grape’ and computation of fruit-to-leaf ratios. The input image **(A)** is segmented to ‘grape’, ‘leaf’ and ‘background’. The segmentation can be used to compute the grape-to-leaf ratio in the 2D image domain **(B)**. A more accurate ratio can be computed in the depth weighted 3D space using the reconstructed surface **(C)**.

The use of dense depth maps enables a scaling of each pixel according to its depth which corresponds to the actual size of the area captured in the pixel. The respective area computation in 3D can increase accuracy of the resulting complex phenotypic data, in comparison to area computation in the 2D image plane. Figure 3 shows a comparison of the average grapes-to-leaf ratio in 2D (pixel) and 3D (actual size). Computing the ratio in 3D results in a 10% decreased ratio compared to the computation in 2D.It can balance out the fact that some leaves are closer to the camera and thus occupy a disproportionally larger area in the 2D image plane than the grapes that are farther away. This effect can also be observed in the Additional file 1 which shows a 3D view of the surface shown in Figure 3C.

Thus, the presented computation of fruit-to-leaf ratios provides an efficient method to objectively evaluate yield efficiency of red grapevine cultivars.

### Statistical evaluation and error analysis

**Figure 4:**
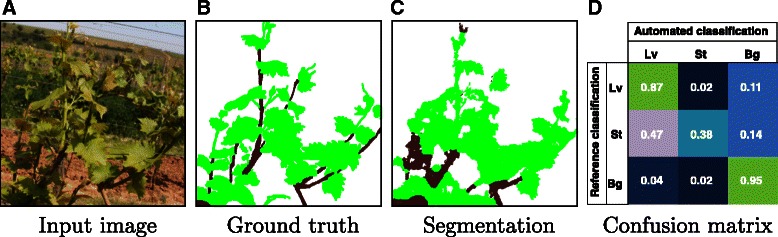
Evaluation of the segmentation results by comparison to ground truth data. A set of 22 images **(A)** was used to compare manually labeled ground truth images **(B)** to the computed segmentation results **(C)**. The confusion matrix **(D)** shows that the proposed method correctly classifies the majority of pixels as ‘leaf’ (Lv), ‘stem’ (St) or ‘background’ (Bg).

For validation of the method, 22 images of the data set (as the example shown in Figure 4A) were manually segmented and used as ground truth (Figure 4B). The ground truth was used for comparison with the computed segmentation results of the algorithm (Figure 4C).

A confusion matrix was generated from the 22 images in order to investigate the precision of the computed classification results (Figure 4D). The matrix represents the relation between actual classifications in rows and predicted classifications in columns, for the three classes ‘leaf’, ‘stem’ and ‘background’. It reveals that the major percentage of all three classes was correctly classified by the automated segmentation algorithm. The best results can be observed for the classes ‘background’ (95% correct classifications) and ‘leaf’ (87%). The segmentation of the class ‘stem’ shows the highest false classification rate with 47% pixels classified as ‘leaf’. A reason for this might be the fact that young branches often have green color and therefore get falsely classified as ‘leaf’. This indicates that the distinction between these two plant organs is not accurate enough when only color information is considered. Additional analysis of geometric information would be required for more accurate classifications, similar to the approaches recently published for point clouds in [28] and dense surfaces in [20,21]. Such an extension might be a promising improvement of the presented method.

**Figure 5:**
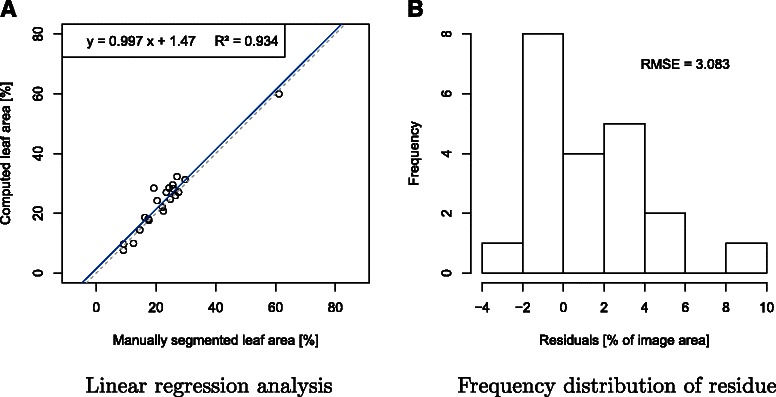
Validation and error analysis with N = 22 test images.**A**. The linear regression analysis shows the difference of leaf area from reference classifications (ground truth) and the computed image segmentations (predicted leaf area). **B**. Frequency distribution of observed residue and the root-mean-square error (RMSE) of the difference between predicted leaf area and ground truth.

The accuracy of the computed leaf area was evaluated using the software R for statistical computing. To this end, the computed leaf area was compared with the ground truth leaf area. Figure 5A shows the linear regression analysis where a linear equation was determined from the segmentation results. The regression analysis showed a determination coefficient of R ^2^=0.93. Further, the estimated regression line (*y*=0.997*x*+1.47) was used to predict the leaf area. The slope of 0.997 implies that the error is not systematical. An error analysis of the computed leaf area was performed by calculation of the frequency distribution of observed residue and the root-mean-square error (RMSE). Figure 5B shows the results of this analysis. Every pixel of the 22 computed classifications was compared to the respective ground truth classification, in order to determine the precision of the developed method. The leaf areas were normalized to a range of 0 to 100 by the size of the image domain. The residuals are given as absolute values. The ground truth reference data was plotted against the computed leaf area and a root-mean-square error of 3.083% was calculated. This implies that the regression line approximately represents the reference data. Furthermore, 68% of the residuals are within a bound of ±2.5 around the mean 1.4, 95% are within a bound of ±3.9 and 99.7% are within a bound of ±7.7.

### Performance and efficiency of the method

To compute globally optimal depth reconstructions, we optimize the stereo problem in a higher dimensional space. This requires the respective amount of memory and run-time. In the applications used in this study, the depth map computation was processed off-line after image capturing, and is thus not time-critical, whereas the image capturing can be processed in real-time. The examples shown in this study were computed on an *Nvidia GeForce GTX Titan* GPU with 6 GB memory. We chose an image resolution of 1024 × 1024 and a depth resolution of 256, because it fits the memory of the GPU. The computation time for this resolution takes about 10 minutes using the *Cuda* programming language for parallel processing.

We use a single camera to capture the stereo image pairs from the grapevines of interest. This makes the image acquisition simple and inexpensive, however the relative camera positions are different for each image pair. In consequence, the depth range is also variable, and needs to be manually adjusted for each image pair. This corresponds to a user input of one value for each image pair, making the method practicable for the applications shown in this study. Furthermore, in 5% of the image pairs used in the experiments, the relative position of the camera capture positions could not be reconstructed. This is usually the case when the distance or orientation angle of the camera positions are too large, and hence not enough corresponding points can be found in the two images. Typically, these images do not have enough overlap, or the images do not contain enough structure. We further observed that wind can cause the plant to move from one image capturing moment to the next. In this case, the depth maps contain incorrect parts and the image acquisition has to be repeated. This problem can be overcome by using two cameras that capture simultaneously as used in [23]. Applying a similar stereo system would be an interesting extension for future work. Then, the relative position of the cameras and the depth range could be calibrated once, which would be an efficient way to save computation time and eliminate the need for user interaction.

## Conclusions

High-throughput field phenotyping of perennial plants like grapevine requires a combination of automated data recording directly in the field and automated data analysis. Using only image data from unprepared fields, the segmentation into foreground (grapevine) and background (field) constituted the major challenge in this study. Especially at the beginning of a growing season, an automated segmentation based on color only is impossible in single field images as very similar color distributions occur in foreground and background. To overcome this problem, most related works either install artificial backgrounds behind the plants or use depth information generated by e.g. 3D laser scanners.

We presented a novel approach for the segmentation of field images to the phenotypic classes ‘leaf’, ‘stem’, ‘grape’, and ‘background’, with a minimal need for user input. In particular, only one free parameter needs to be manually adjusted for each input image pair, which corresponds to the depth segmentation of foreground and background. The method is based on RGB image pairs, which requires just a low-cost standard consumer camera for data aquisition. We showed robust background subtraction in field images by the use of dense depth maps. This avoids the necessity of costly 3D sensor techniques or elaborate preparation of the scene. We further showed how the method allows for objective computation of canopy dimensions (digital leaf area) which enables the monitoring and characterization of plant growth habits and computations of fruit-to-leaf ratios.

Future plans for the application of this approach include the installation of a stereo camera system where the cameras are mounted with fixed position to each other, for a standardized image acquisition setup. Thus, the depth parameter for the image segmentation can be set constant, in order to reduce the need for user interaction. Furthermore, refinements of the method are possible, including an automated detection of wires in the images and other objects that appear in the foreground but do not belong to the plant. The consideration of geometric information to distinguish between leaves and stem would be interesting to investigate. This might be important in order to reduce false positive classifications and thus, enhance the accuracy of the method.

The presented method provides a promising tool for high-throughput image-based phenotyping in vineyards. The ability to accurately and quickly monitor phenotypic plant growth, particularly after bud burst, facilitates an improvement to vineyard management, and the early detection of growth defects. Furthermore, the automated analysis of phenotypic traits like fruit-to-leaf ratios, that were usually acquired manually in the past, allows for processing of large data sets of plants. Thus, the method might provide a step towards the automated validation or determination of optimal fruit-to-leaf ratios from a large variety of plants and cultivars.

## Methods

The workflow of the proposed image-based phenotyping approach is shown in Figure 1: First, a stereo image pair is captured in a vineyard with a standard RGB camera. These image pairs are rectified in a pre-processing step in order to transform the image planes to a normalized case (Figure 1A,B). The rectified images facilitate the computation of dense depth maps (Figure 1C). Furthermore, one of the two images is classified by a color classifier enhancing green plant organs (Figure 1D), and image edges are detected in order to preserve fine-scaled structures of the plant (Figure 1E). These features are used to compute a segmentation of the image domain to the phenotypic classes ‘leaf’, ‘stem’ and ‘background’ (Figure 1F). The resulting segmentations are applicable for phenotypic computations like the quantification of visible 3D leaf areas.

### Field experimental setup

The method was validated and tested with a database of 90 images of grapevine plants, captured at five different dates during the 2011 season. For digital phenotyping, we chose images from genotypes with similar phenology, i.e. similar time of bud burst and flowering. Therefore, images of ‘Riesling’, ‘Villard Blanc’ and two breeding lines were selected for further investigation.

#### Experimental site

The experiments involved plants of the *Vitis vinifera* cultivars ‘Riesling’ and ‘Villard Blanc’ (three plants per cultivar) as well as two breeding lines (F1 generation of the crossing Gf.Ga.47-42 × ‘Villard Blanc’) at the experimental vineyard of Geilweilerhof located in Siebeldingen, Germany (N 49°21.747, E 8°04.678). For the breeding lines only one plant per genotype is available. Bud burst at BBCH 10 of all selected genotypes was detected at the 100th day of the year 2011, and the flowering began at the 145th day of the year 2011. Hence, the selected genotypes showed similar phenology. ‘Villard Blanc’ displayed a slow growth rate (OIV 351 class 3) whereas ‘Riesling’ displayed a medium growth rate (OIV 351 class 5) [unpublished data].

#### Image acquisition

A single-lens reflex (SLR) camera (Canon EOS 60D) was used to capture RGB images in the vineyard. The SLR camera was fixed with variable height mounting above ground level (1.00 – 1.30 m) in the middle of a wheeled carrier vehicle (Fetra GmbH, Hirschberg, Germany). Image acquisitions were carried out in front of the grapevine plants with a distance of at least 1 m by dragging this platform between the grapevine rows as described in [6]. Due to the limited space between two rows this enables a stable distance to the plants and hence allows for comparison of images taken at different time points. The image pairs were captured in the field under natural illumination conditions with manually controlled exposure. No predefined exposure time was used. Image pairs of each plant were captured from different vantage points, in a way that the depicted areas are overlapping. The height of the camera above ground level was adapted in order to standardize the image acquisition. The woody cane of the grapevines was used as reference for the captured grapevine section (the cane or parts of the cane must be visible in the image). This implies that the center of the grapevine is acquired.

### Image rectification

In a pre-processing step, the captured images are rectified, using the software of [26] for identification of key points and the software of [29] for the subsequent rectification transformation. In rectified images, epipolar lines are parallel which simplifies the subsequent computation of depth maps. Rectification implies a reprojection of two images, such that both projected images lie in the same plane and geometrical distortions are corrected. To rectify an image pair, the camera parameters are calibrated, i.e. lens distortion and relative camera positions are estimated. With these parameters homographies are computed that facilitate the image transformation.

### Dense depth reconstruction

Depth reconstruction aims at inferring 3D structure from a pair of rectified 2D images, in the following denoted by $I_{1},I_{2}:\Omega \rightarrow \mathbb {R}^{3}$. The images are defined in the image domain $\Omega \subseteq \mathbb {R}^{2}$. We represent 3D information with a dense depth map $d:\Omega \rightarrow \mathbb {R}$ which assigns to each pixel *x* of the reference image *I*_1_ the distance (also denoted by the *depth*) of the respective 3D point to the camera.

Depth can be computed from *disparity*, which is the displacement of image locations of an object point. In order to deduce disparity from the rectified images, pixel pairs that show the same object point have to be identified. Given the images $I_{1},I_{2}:\Omega \rightarrow \mathbb {R}^{3}$, we compute a dense disparity map *v*:*Ω*→*Γ*:=[0,*γ*_max_] by minimizing a higher dimensional variable *ϕ*:*Ω*×*Γ*→[0,1] as in [30,31]. Optimization in the product space *Ω*×*Γ* of the image domain *Ω* and the range of disparities *Γ* allows for a convex formulation of the stereo reconstruction problem, and therefore enables global optimization. Thus, the disparity map *v* is computed by integration over *ϕ*:

(1) $$ v(x) = \int_{\Gamma} \phi \,\mathrm{d}\gamma,   $$

and *ϕ* is a minimizer of

(2) $$ \begin{aligned} &\min_{\phi\in C} \left\lbrace \int_{\Omega\times\Gamma} |I_{1}(x)-I_{2}(x+\gamma)| | \partial_{\gamma}\phi| \,\mathrm{d}x\mathrm{d}\gamma \right.\\ &\,\,\qquad+ \left.\lambda \int_{\Omega\times\Gamma} | D\nabla(\phi)| \,\mathrm{d}x\mathrm{d}\gamma \right\rbrace, \end{aligned}   $$

with

(3) $$ C = \left\lbrace \phi:\Omega\times\Gamma\rightarrow[0,1] \,:\, \phi(x,0) = 1,\, \phi(x,\gamma_{\text{max}}) = 0\right\rbrace.   $$

The first term in (2) is the data fidelity term which measures point-wise color differences between the two images. The second term is the regularizer term, weighted by a smoothness parameter $\lambda \in \mathbb {R}$. The *L*_1_ norm in the regularizer yields piecewise smooth solutions while preserving edges. We further weight the gradient norm with an anisotroy tensor *D* which serves as an edge enhancing function, in order to preserve the fine-scaled structures of the plants. An visual representation of *D* is shown in Figure 1E, where the color of each pixel encodes the direction of the local image gradient and the intensity encodes the length of this vector. The optimization problem can be globally optimized as described in [31] while the constraint set *C* ensures that the global minimum of 2 is not the trivial solution.

Disparity maps give measurements in pixel units, while depth is measured in absolute scale. The depth *d* is proportional to the inverse of the disparity *v* and is computed by

(4) $$ d(x) = \frac{f b}{v(x)},  $$

where *f* is the focal length of the camera and *b* is the baseline, i.e. the distance between the two camera capturing positions.

### Image segmentation using color and depth

Besides computing depth information from images, the images are segmented with respect to the color and depth information. Image segmentation is the partitioning of the image domain into meaningful regions, i.e. each pixel in the image domain *Ω* gets assigned a label *l*∈*L*={1,…,*n*}. We segment the image domain to *n*=3 regions corresponding to ‘stem’, ‘leaf’ and ‘background’. An example of a segmented image is shown in Figure 1F, where green regions represent the class ‘leaf’, brown regions ‘stem’, and white regions ‘background’. To compute the segmentation, we use the method of [32], using the following two classifiers *f*_depth_ (5) and *f*_color_ (6):

First, the reconstructed depth map *d* gives information about location of the foreground and background. We use the following function

(5) $$ f_{\text{depth}}(x) = d(x) - c_{\text{depth}},   $$

to implement the assumption that the background is farther away from the camera capturing position than the foreground plant. An example for *f*_depth_ is shown in Figure 1C, where the color encodes the depth of each pixel, ranging from ‘red = near’ to ‘blue = far’. The free parameter $c_{\text {depth}}\in \mathbb {R}$ is dependent on the maximum depth that the foreground plant can take. It can be assumed constant for standardized image capturing processes, or if distances of the camera capturing positions and plants vary only in a specified range. In the experiments shown in this paper *c*_depth_ was adjusted manually for each image pair.

Second, the foreground is classified as ‘leaf’ or ‘stem’, using the color information of the reference image *I*_1_:

(6) $$ f_{\text{color}}(x) = I_{1}^{\text{green}}(x)-I_{1}^{\text{blue}}(x) - c_{\text{color}}.   $$

Subtracting the blue color channel *I* ^blue^ from the green color channel *I* ^green^ yields a robust classifier for vegetation [19]. An example for *f*_color_ is shown in Figure 1D, where green regions represent high function values and blue regions represent low values. The free parameter $c_{\text {color}}\in \mathbb {R}$ is dependent on the type and stadium of the plant, as well as the prevalent illumination and weather conditions of the scene. In the experiments shown in this paper *c*_color_=20 was chosen by experiments, for RGB values ranging from 0 to 255.

The implementation of the additional class ‘grape’ classifies red and blue colored pixels as grapes, enabling the computation of fruit-to-leaf ratios.

### Computation of leaf surface areas

The 3D digital leaf surface area is computed from the segmented images and the depth maps by weighting the pixel sizes according to their depth. The area of region *Ω*_i_ is computed from the segmentation *u* and depth map *d* by:

(7) $$ \text{Area}(\Omega_{i}) = \int_{\Omega} d(x)^{2} u_{i}(x) \,\text{dx},  $$

where the size of a pixel is computed as *d*(*x*)^2^, normed by the focal length *f* of the camera, as in [33]. The weighting balances out the fact that due to projection in the image capturing process, the depicted objects in the image do not appear according to their actual size – objects that are near the camera occupy a larger region in the image than parts that are farther away.

## Additional file

Additional file 1
**Shows a video of the reconstructed 3D grapevine surface from Figure** 3C.
